# Dynamics of Soil Respiration in Alpine Wetland Meadows Exposed to Different Levels of Degradation in the Qinghai-Tibet Plateau, China

**DOI:** 10.1038/s41598-019-43904-1

**Published:** 2019-05-16

**Authors:** Zhongfei Li, Jixi Gao, Linqin Wen, Changxin Zou, Chaoyang Feng, Daiqing Li, Delin Xu

**Affiliations:** 1 College of Ecology and Environment, Southwest Forestry University, Kunming, 650224 Yunnan China; 2 Nanjing Institute of Environmental Sciences, Ministry of Ecology and Environment, Nanjing, 210042 Jiangsu China; 3 Chinese Research Academy of Environmental Sciences, Beijing, 100012 China; 4 State Environmental Protection Key Laboratory of Regional Eco-process and Function Assessment, Beijing, 100012 China

**Keywords:** Carbon cycle, Ecosystem ecology

## Abstract

The effects of degradation of alpine wetland meadow on soil respiration (Rs) and the sensitivity of Rs to temperature (Q_10_) were measured in the Napa Lake region of Shangri-La on the southeastern edge of the Qinghai-Tibet Plateau. Rs was measured for 24 h during each of three different stages of the growing season on four different degraded levels. The results showed: (1) peak Rs occurred at around 5:00 p.m., regardless of the degree of degradation and growing season stage, with the maximum Rs reaching 10.05 μmol·m^−2^·s^−1^ in non-degraded meadows rather than other meadows; (2) the daily mean Rs value was 7.14–7.86 μmol·m^−2^·s^−1^ during the mid growing season in non-degraded meadows, and declined by 48.4–62.6% when degradation increased to the severely degraded level; (3) Q_10_ ranged from 7.1–11.3 in non-degraded meadows during the mid growing season, 5.5–8.0 and 6.2–8.2 during the early and late growing seasons, respectively, and show a decline of about 50% from the non-degraded meadows to severely degraded meadows; (4) Rs was correlated significantly with soil temperature at a depth of 0–5 cm (p < 0.05) on the diurnal scale, but not at the seasonal scale; (5) significant correlations were found between Rs and soil organic carbon (SOC), between biomass and SOC, and between Q_10_ and Rs (p < 0.05), which indicates that biomass and SOC potentially impact Q_10_. The results suggest that vegetation degradation impact both Rs and Q_10_ significantly. Also, we speculated that Q_10_ of alpine wetland meadow is probable greater at the boundary region than inner region of the Qinghai-Tibet Plateau, and shoule be a more sensitive indicator in the studying of climate change in this zone.

## Introduction

Research indicates that atmospheric CO_2_ concentrations rose from 280 ppm in 1975 to 397 ppm in 2014^1^, and will potentially rise to 500–1000 ppm by 2100 if no corrective actions are taken^2,3^. Atmospheric CO_2_ concentrations are strongly influenced by carbon flux in terrestrial ecosystems, especially by soil respiration (Rs) processes, which can emit ~120 Pg of carbon to the atmosphere per year^4^. This rate is higher than carbon emissions from anthropogenic fossil fuel combustion^5,6^. In terrestrial ecosystems, the amount of carbon emitted from Rs processes is second only to the amount of carbon fixed by gross primary productivity (GPP) and is even more than the carbon uptake by net primary productivity (NPP) in certain situations^7–9^.

Rs is a key component of carbon flux in the global carbon cycle and a potential indicator of ecosystem metabolism^10,11^. It can also be used to estimate belowground carbon allocation^12^, and to reveal the processes and mechanisms of carbon sources and sinks on regional and global scales. More precisely, Rs can be used to predict future atmospheric CO_2_ concentrations and the degree and rate of climate change^4,13^. However, due to the high temporal and spatial heterogeneity of Rs, it can only be accurately measured directly within each specific location, which makes it difficult to simulate, predict, and assess the spatial and temporal dynamics of Rs at global and regional scales^14^, and to identify how these dynamics contribute to climate change^15,16^. Therefore, quantitative field measurement for Rs in various ecosystem types is still urgently needed in research of global carbon cycle, which will contribute to reducing uncertainly when quantifying ecosystem carbon sequestration^17^.

Wetland soils, which constitute only 2–3% of global land area, store a disproportionate amount of global soil carbon (18–30% of the total 1550 Pg of soil carbon)^18,19^. However, data on soil carbon dynamics in wetland regions is scarce^20^. For example, there are only 135 records for wetlands among a total of 3821 records in the global Rs database (SRDB version 20100517)^16,20^. China contains about 4% wetlands, making it one of the richest countries in terms of wetland resources in the world^21^. Wetlands are typically carbon sinks^22–25^, because the anaerobic environment, high productivity, and low soil temperature (Ts) can tend to reduce decomposition and promote peat formation^26–32^.

Some studies identify the alpine wetland meadows of the Qinghai-Tibetan Plateau (QTP) in China as a huge organic carbon sink that is highly sensitive to global climate change^33,34^. However, other studies classify the QTP wetland meadows as a carbon source^35,36^. It is therefore important to resolve this discrepancy by examining the influence of different types or conditions of aboveground vegetation on soil carbon emission^37^. There is an urgent need to understand the carbon exchange processes occurring in alpine wetland meadows^34,38^.

The Shangri-La region, located in northwestern Yunnan Province at the eastern edge of the QTP, lies in the Hengduan Mountains^39,40^. It contains rich biodiversity and serves as an important ecosystem service spillover region. Numerous plateau lakes and alpine wetland ecosystems are distributed throughout the region, with characteristics typical of a low-latitude and high-altitude geographical environment. As global climate change and human adaptation progress, however, these alpine wetland ecosystems face unprecedented threats, including drainage transformation, reclamation, tourism development, and shortage of water resources. A large number of seasonally-flooded alpine swamp meadows have begun experiencing long-term exposed to water, which has turned these swamp meadows into alpine wetland meadows or alpine meadows and caused shifts in community composition and structure and the physical and chemical properties of soil and environmental conditions. Constant disturbances from human activities, such as overgrazing of livestock and trampling by tourists, have caused a great number of wetland meadows to further degrade. Ultimately, alpine wetland meadow degradation leads to changes in carbon budgets and the balance of ecosystems^29,41,42^.

The boundary region of the QTP may exhibit a more sensitive response to climate change than other regions^43^. In the past few decades, the QTP has experienced more rapid warming than other regions of the world^44–46^. Many studies on carbon flux in the QTP have been conducted^37,47–49^, including studies in alpine meadows, and wetland meadows^36,50–53^, but almost all of these studies have been conducted in the hinterland region of the QTP instead of in the boundary region. Research in the Shangri-La region on the southeastern edge of the QTP is almost nonexistent. The dual effects of human activities and climate change on carbon flux in the natural ecosystems of this region are still unknown.

For this study, we selected alpine wetland meadow, which is one of three grassland types (including alpine meadow, alpine shrubland meadow, and alpine wetland meadow) in QTP, and is perennially exposed to water, in the Napa Lake region of Shangri-La, located on the southeastern edge of the QTP. Within the alpine wetland meadows, we identified four levels of degradation severity, based on fencing and grazing, tourism trampling, vegetation coverage, and aboveground biomass. Rs was measured for 24 h within plots of different degradation severities during the early, middle, and late stages of the growing seasons in 2014 and 2015. The objectives of this study were: (1) to understand the dynamic and variable mechanism of Rs in differently degraded alpine wetland meadows at diurnal, seasonal, and inter-annual time scales; (2) to reveal the effects of vegetation degradation on Rs in alpine wetland meadows; This paper provides a better understanding of the effects of human activities on carbon cycling between land and atmosphere in the QTP region.

## Results

### Thirty-year changes in temperature and precipitation in the study area

**Figure 1:**
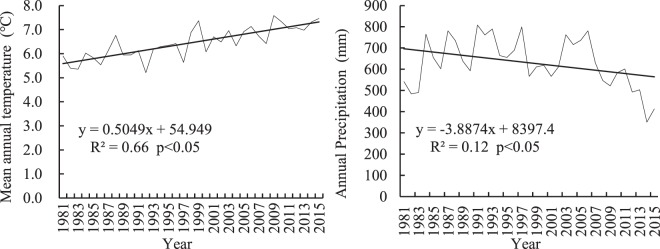
Temperature and precipitation from 1981–2015 at Napa Lake region of Shangri-La.

Figure 1 shows that the average annual temperature in the study region rose from 5.9 °C in 1981 to 7.5 °C in 2015, for a total increase of 1.6 °C. The average temperature increase between 1990 and 2000 was 0.37 °C greater than the average temperature increase between 1981–1990, and the average increase between 2000 and 2010 was 0.60 °C greater than that between 1990 and 2000, suggesting that the size of the temperature increase has grown over time.

Precipitation presents a decreasing trend (p < 0.01) (Fig. 1). The precipitation decline mainly occurred after 2005. Average annual precipitation between 2006 and 2015 was only 542.4 mm, which is much lower than the annual averages between 1981 and 1990 (628.5 mm) and between 1990 and 2000 (696.6 mm) (Fig. 1).

### Vegetation conditions in alpine wetland meadows impacted by different levels of degradation

Table 1 shows that vegetation coverage, aboveground biomass, and LAI were significantly lower in the SDM than in the NDM (p < 0.05); vegetation coverage and biomass were 50% lower and LAI was 80% lower. Vegetation coverage and aboveground biomass were about 40% and 75% lower in the SDM than the NDM. This suggests that alpine wetland meadow degradation results in a significant decrease in the condition of aboveground vegetation (p < 0.05).

**Table 1:** Vegetation conditions in alpine wetland meadows impacted by different levels of degradation.

Degraded Levels	Vegetation Coverage (%)	Aboveground Biomass (g·m^−2^)	LAI	Dominant Species	Human Activity
NDM	95.0 ± 1.6a	304.8 ± 14.9a	2.4 ± 0.2a	*Blysmus sinocompressus*, *Carex muliensis*, *Poa szechuensis*	Fenced over 20 years, no grazed, reaped and tourism disturbance
LDM	84.3 ± 3.3b	246.2 ± 3.0b	1.8 ± 0.1b	*Blysmus sinocompressus*, *Carex muliensis*	Fenced over 10 years, reaped per year, no grazed and tourism disturbance
MDM	66.7 ± 2.4c	101.7 ± 5.7c	0.9 ± 0.1c	*Potentilla anserina, Pedicularis longiflora*	Grazed, but no fenced and tourism disturbance
SDM	50.0 ± 4.1d	78.5 ± 4.5d	0.4 ± 0.1d	*Potentilla anserina*	Grazed and tourism disturbance, but no fenced

Note: (1) The data is mean value ± SD. Different letters within a line indicate a significant difference between mean values (p < 0.05) for different levels of degradation. (2) NDM, LDM, MDM, and SDM represent non-degraded meadow, lightly-degraded meadow, moderately-degraded meadow and severely-degraded meadow respectively.

### SOC in alpine wetland meadows impacted by different levels of degradation

Table 2 shows that the carbon content of vegetation in alpine wetland meadows decreased significantly (p < 0.05) with increasing degradation. For example, carbon content was approximately 15.8% lower in the SDM than in the NDM.

**Table 2:** SOC of alpine wetland meadows impacted by different levels of degradation.

Degraded Levels	Carbon Content of Vegetation (g·kg^−1^)	SOC in Different Soil Layer (g·kg^−1^)				
		0–10 cm	10–20 cm	20–30 cm	30–40 cm	40–50 cm
NDM	466.9 ± 12.1a	47.0 ± 0.9 aA	25.8 ± 2.0aB	18.1 ± 0.4aC	14.7 ± 1.1aC	7.8 ± 0.7aD
LDM	457.7 ± 7.7ab	33.7 ± 0.9bA	20.1 ± 0.6bB	14.7 ± 0.5bC	13.7 ± 0.3aC	6.4 ± 0.5aD
MDM	443.4 ± 6.4b	25.6 ± 1.5cA	17.0 ± 0.8bB	11.4 ± 0.4bC	6.9 ± 0.4bD	6.9 ± 0.6aD
SDM	392.9 ± 7.6c	20.5 ± 0.6cA	10.0 ± 0.6cB	6.5 ± 0.9cC	5.0 ± 0.3bC	6.8 ± 0.6aC

Note: (1) The data is mean value ± SD. Different lowercase letters within a line indicate a significant difference between mean values (p < 0.05) for different levels of degradation. Capital letters within a row indicate a significant difference between soil layers (p < 0.05). (2) NDM, LDM, MDM, and SDM represent non-degraded meadow, lightly-degraded meadow, moderately-degraded meadow and severely-degraded meadow respectively. (3) SOC is soil organ carbon.

SOC content in the 0–30 cm soil layer declined significantly between the NDM and SDM levels of degradation (p < 0.05). Specifically, SOC declined by 56.4% in the 0–10 cm soil layer, 61.2% in the 10–20 cm layer, and 64.1% in the 20–30 cm layer. But there was no significant difference in SOC content (p > 0.05) between the NDM and LDM levels in the 30–40 cm soil layer, nor among any of the degradation levels in the 40–50 cm soil layer (p > 0.05) (Table 2).

### Diurnal and seasonal Rs variation within different levels of degradation in 2014 and 2015

**Figure 2:**
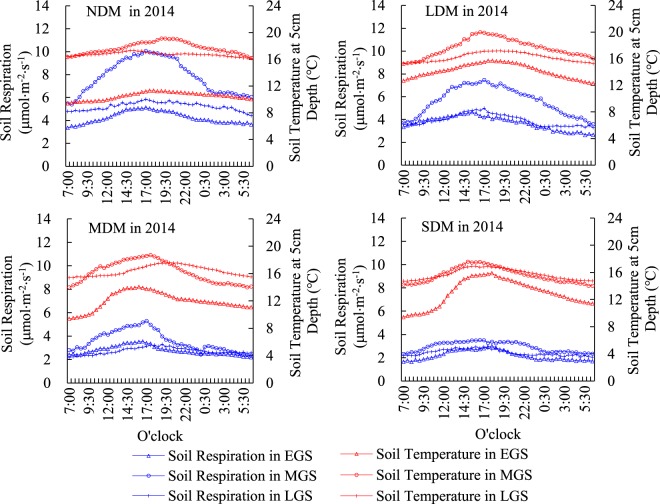
Diurnal and seasonal variations of Rs and Ts in plots impacted by different levels of degradation in 2014. Note: 1. NDM, LDM, MDM, and SDM represent non-degraded meadow, lightly-degraded meadow, moderately-degraded meadow and severely-degraded meadow respectively. 2. EGS, MGS and LGS represent early growing season (May), mid growing season (July), and late growing season (September) respectively.

**Figure 3:**
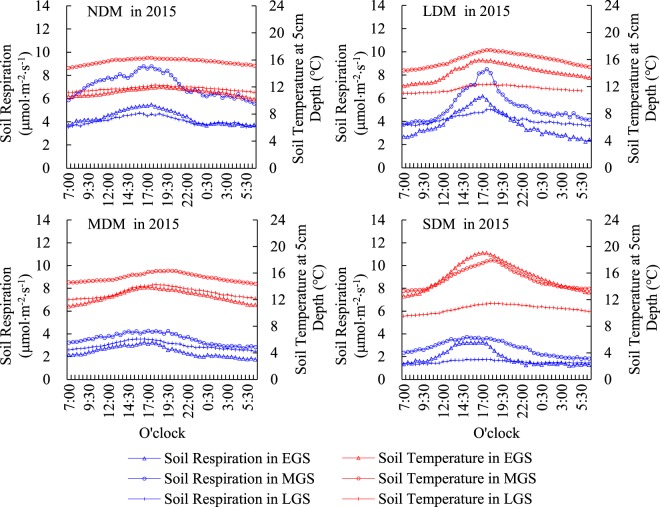
Diurnal and seasonal variations of Rs and Ts in plots impacted by different levels of degradation in 2015. Note: 1. NDM, LDM, MDM, and SDM represent non-degraded meadow, lightly-degraded meadow, moderately-degraded meadow and severely-degraded meadow respectively. 2. EGS, MGS and LGS represent early growing season (May), mid growing season (July), and late growing season (September) respectively.

**Figure 4:**
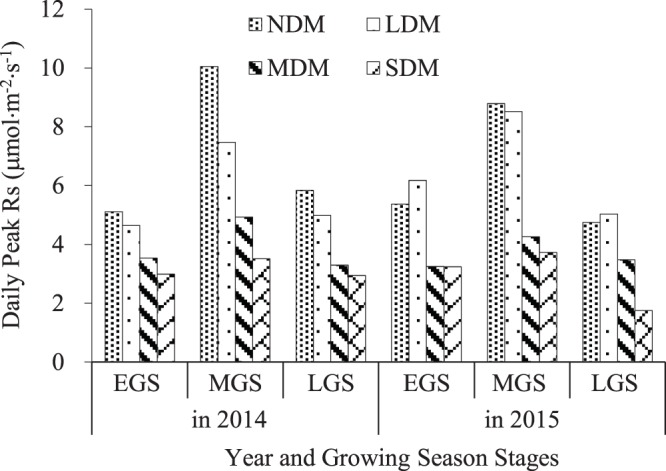
Daily peak Rs at different levels of degradation in alpine wetland meadows in 2014 and 2015.

Figures 2 and 4 show the diurnal and seasonal variations in Rs and Ts in plots impacted by different levels of degradation in 2014 and 2015. Rs in all plots showed a single peak in the diurnal analysis, which occurred at around 5:00 pm. Beginning at 7:00 a.m., Rs fluctuated in an increasing direction from 7:00 am until the peak, and then fluctuated in a decreasing direction until 6:30 am the next morning. Peak Ts values appeared between 3:00 p.m.–8:00 p.m. in every plot (Figs 2 and 3).

As for seasonal variations, Ts were highest during the MGS and were basically equivalent during the EGS and LGS at all levels of degradation in both 2014 and 2015 (Figs 2 and 3). Diurnal and seasonal variations in Rs and Ts within the different degraded plots were similar between 2014 and 2015.

### Variation in daily peak Rs at different levels of degradation

Figure 4 shows that peak Rs decreased significantly (p < 0.05) with increasing degradation. Peak Rs ranged from 4.64–10.05 μmol·m^−2^·s^−1^ in the NDM and LDM plots, but only 1.75–4.93 μmol.m^−2^ s^−1^ in the MDM and SDM plots. In 2015, peak Rs was 5.37 μmol·m^−2^·s^−1^ during the EGS, 8.78 μmol·m^−2^·s^−1^ during the MGS, and 4.74 μmol·m^−2^·s^−1^ during the LGS in the NDM. During the same stages of the growing season in the SDM, peak Rs was lower by 39.7% during the EGS, 57.6% during the MGS, and 63.1% during the LGS.

Peak Rs was highest during the MGS. Peak Rs was lower, but similar, during the EGS and LGS. Peak Rs values during the MGS were about 44.0% higher than during the other two growing season stages in the NDM and about 35.0% higher in the LDM, but only 26.0% higher in the MDM and 24.2% higher in the SDM (Fig. 4).

### Daily mean Rs value at different levels of degradation

**Figure 5:**
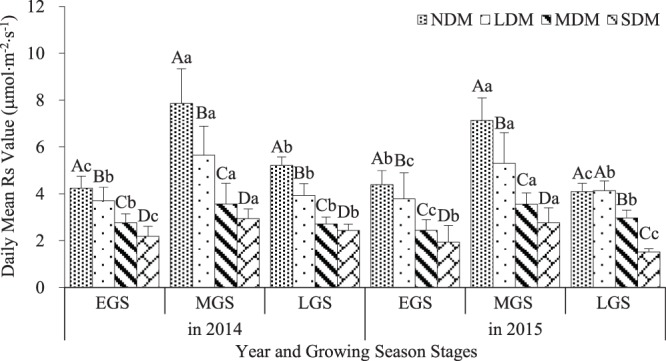
Daily mean Rs value at different levels of degradation in alpine wetland meadows in 2014 and 2015. Note:Different lowercase letters indicate significant differences between daily mean Rs values (p < 0.05) at different levels of degradation. Capital letters indicate significant differences between growing season stages in the same year. Bars indicate SE of mean.

Figure 5 shows the daily mean Rs values at different levels of degradation. Overall, the values decreased significantly as the level of degradation increased (p < 0.05). The daily mean Rs value reached 4.24 μmol·m^−2^·s^−1^ during the EGS, 7.86 μmol·m^−2^·s^−1^ during the MGS, and 5.22 μmol·m^−2^·s^−1^ during the LGS in the NDM in 2014. These values were 48.4%, 62.6%, and 53.2% lower, respectively, in the SDM. Daily mean Rs values in the NDM in 2015 were 4.39 μmol·m^−2^·s^−1^ during the EGS, 7.14 μmol·m^−2^·s^−1^ during the MGS, and 4.09 μmol·m^−2^·s^−1^ during the LGS, which, similar to 2014, were higher than the daily mean Rs values in the SDM by 55.8%, 61.2%, and 62.8%, respectively.

The daily mean Rs value during the MGS was approximately 40.0% higher than during the other two growing season stages in the NDM, about 28.8% in the LDM, but only 23.6% and 29.3% in the MDM and SDM, respectively (p < 0.05) (Fig. 5).

### Correlation of Rs and Ts

**Figure 6:**
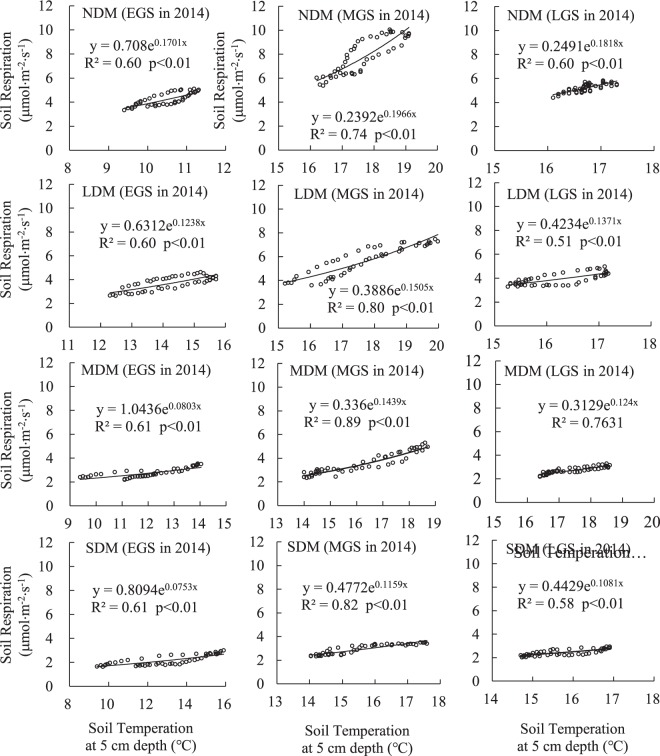
Exponential correlation of Rs and Ts at the 0–5 cm soil depth in 2014.

**Figure 7:**
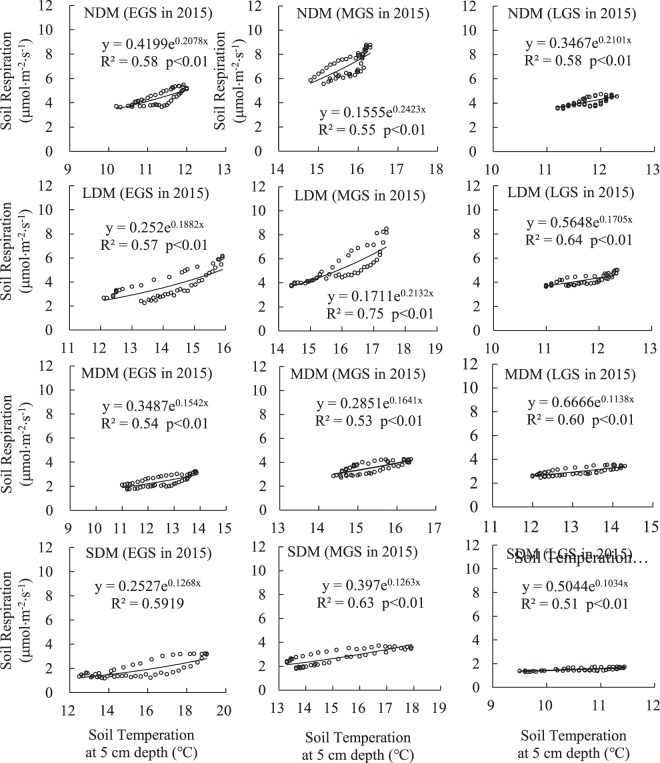
Exponential correlation of Rs and Ts at the 0–5 cm soil depth in 2015.

Figures 6 and 7 show the power exponential curve relationship between Rs and Ts at the 0–5 cm soil depth within different levels of meadow degradation and during different stages of the growing season in 2014 and 2015. Almost all correlation coefficients (R^2^) were above 0.5, and most of them were above 0.6. All power exponents passed the significance test (p < 0.01).

### 3.8. Q_10_ values for different levels of degradation

**Figure 8:**
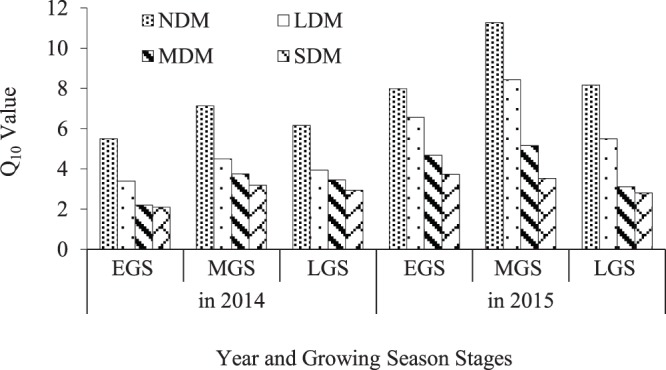
The Q_10_ values for meadows with different levels of degradation in 2014 and 2015.

Figure 8 shows the variation characteristics of Q_10_ in different levels of degradation during different stages of the growing season. The Q_10_ in the NDM reached 5.5, 7.1, and 6.2 during the EGS, MGS, and LGS, respectively, in 2014, but decreased from the NDM levels by more than 30%, 40%, and 50% in the LDM, MDM, and SDM, respectively. Q_10_ was 8.0, 11.3, and 8.2 in the NDM during the EGS, MGS, and LGS, respectively, in 2015; these values were slightly higher than in 2014, but the variation between the two years was very similar.

The Q_10_ values during the MSG were higher than during the ESG and LSG. There was no significant difference between the ESG and LSG Q_10_ values.

### Correlations among vegetation biomass, SOC, Rs, and Q_10_

Table 3 shows the correlations among aboveground biomass, SOC, daily mean Rs value, and Q_10_ in alpine wet meadows impacted by four different levels of degradation. Aboveground biomass correlated significantly with SOC at the 0–10 cm and 30–40 cm soil depths (p < 0.05), but not at other soil depth (p > 0.05), and with Q_10_ (p < 0.05).

**Table 3:** Correlations among aboveground biomass, SOC, Rs, and Q_10_ in 2014 and 2015.

	Biomass	SOC1	SOC2	SOC3	SOC4	SOC5	Rs1	Rs2	Q_10_1	Q_10_2
Biomass	1									
SOC1	0.96*	1								
SOC2	0.92	0.96*	1							
SOC3	0.94	0.96*	0.99**	1						
SOC4	0.99*	0.92	0.91	0.94	1					
SOC5	0.45	0.68	0.57	0.51	0.33	1				
Rs1	0.90	0.99**	0.94*	0.95	0.95	0.62	1			
Rs2	0.99*	0.99**	0.96*	0.97*	0.95*	0.61	0.99**	1		
Q_10_1	0.98*	0.98*	0.92	0.9	0.83	0.79	0.97*	0.99*	1	
Q_10_2	0.98*	0.98*	0.97*	0.98*	0.97*	0.53	0.96*	0.99**	0.93	1

Notes: *Correlation is significant at the 0.05 level (2-tailed). **Correlation is significant at the 0.01 level (2-tailed). Biomass is the aboveground vegetation biomass in MGS. SOC1 is at 0–10 cm depth. SOC2 is at 10–20 cm depth. SOC3 is at 20–30 cm depth. SOC4 is at 30–40 cm depth. SOC5 is at 40–50 cm depth. Q_10_1 is in MGS of 2014, and Q_10_2 is in MGS of 2015. Rs1 is daily mean Rs value during MGS of 2014, and Rs2 is daily mean Rs value during MGS of 2015.

SOC at the 0–10 cm soil depth correlated significantly with Rs and Q_10_ (p < 0.05). At the 10–20 cm soil depth, SOC and Rs correlated significantly (p < 0.05), as did Rs and Q_10_ (p < 0.05).

## Discussion

### The impacts of degradation on Rs in a Napa Lake alpine wetland meadow

Aboveground vegetation degradation has an important effect on Rs^54,55^. In this study, as the severity of degradation in an alpine wetland meadow increased from NDM to SDM, the Rs rate decreased significantly to about 50% of its original rate (Fig. 5). In addition, aboveground biomass, LAI, and SOC also declined significantly (Tables 1, 2).

Related studies have shown that aboveground biomass is an important modulating factor of Rs^56–58^. In this study, the positive correlation between Rs (in 2015) and aboveground biomass was significant (Table 3), and the positive correlation between Rs and SOC was significant (Table 2). Moreover, the SOC of the top soil layers correlated directly with aboveground biomass (Table 3). Degradation decreases aboveground biomass, which in turn reduces the activities of biological processes of roots and soil^59,60^, and SOC content was observed to decrease synchronously (Tables 2, 3). These direct and indirect effects can ultimately decrease the Rs rate.

Alpine wetland meadows that are perennially exposed to water are typical ecological systems in the Napa Lake region of Shangri-La. Pervasive and long-term disturbances from grazing have caused most of these alpine wetland meadows to become degraded. Grazing has heavily impacted Rs rates^61^ by affecting soil nutrients^62,63^, Ts^64,65^, and aboveground vegetation^66,67^. Meanwhile, other study conduced in this region found that grazing did not significantly soil respiration^68^. Different findings maybe leaded by different vegetation types here. Anyhow, in recent years, frequent trampling from tourism activities in the region has become a more important factor leading to severe degradation of alpine wetland meadows. Together, these human activities have caused serious degradation of alpine wetland meadows, which has disrupted regional carbon balances.

### Sensitivity of Rs to temperature, and variation of Q_10_

Ts is one of the most important factors governing Rs processes on different spatial-temporal scales^69–76^, but much uncertainty remains regarding the influence of other factors on Rs^77–80^. In our study, Rs displayed a significant exponential correlation with Ts on the scale of diurnal variation (Figs 6 and 7), but no significant correlation at the seasonal scale. In contrast, seasonal fluctuations in Rs correlated consistently with seasonal fluctuations in vegetation biomass at every level of degradation in this study (Table 1, Fig. 5). Thus, some researchers have concluded that temperature does not adequately account for all Rs variations^76,81,82^, and that vegetation is also key factor influencing Rs on a seasonal scale^83,84^.

The Q_10_ value, which is the amount that Rs increases with each 10 °C rise in temperature, has commonly been used to assess the sensitivity of Rs to temperature across a variety of ecosystem types and climatic zones^72,85–87^. In this study, we found that the Q_10_ of Rs declined as degradation severity increased in an alpine wetland meadow system (Fig. 8), and that the Q_10_ showed a significant direct correlation with SOC (p < 0.05) and with aboveground biomass (Table 3). These results, which appeared in different seasons and years, are similar to those of other works^11,88^ who studied the alpine meadows of Haibei in the QTP. These results suggest that vegetation degradation can directly reduce the sensitivity of Rs to Ts in the alpine wetland meadows of Napa Lake in Shangri-La.

More and more evidence shows that Q_10_ represents a combination of several influencing factors^89,90^, including biotic and abiotic factors^89,91–93^. In this study, the Q_10_ value was higher during the MGS than during the EGS or LGS (Fig. 8), which is consistent with the seasonal dynamics of aboveground biomass and Rs (Table 1, Fig. 5). A similar seasonal variation pattern in Q_10_ was observed by^94^ in their study of an alpine meadow in Haibei, QTP.

Overall, whether on a time scale of different seasons or different severities of vegetation degradation, the aboveground vegetation condition exerts a significant and decisive influence on Q_10_ in this study.

### Values of Rs and Q_10_ based on transverse comparison with other studies in Shangri-La

Rs is the rate of CO_2_ release from the soil to the atmosphere, and Q_10_ is the sensitivity of Rs to temperature changes. Comparatively high Rs rates indicate relatively high vegetation activity^54,83^, decomposition rates of soil organic matter^65,95^, SOC content^72^, soil microbial biomass^57,68^, soil microbial activity^96,97^, and soil moisture^75,76,80,98^, etc. However, these factors, which are known to increase Rs, are sensitive to shifts in climate conditions, such as rising temperatures^13,85^. Meanwhile, Q_10_ is a major source of uncertainty in assessing carbon budgets using carbon cycle models^99,100^, because differences in Q_10_ among different ecosystems have been left out of many terrestrial carbon models^101,102^. Therefore, it is important to identify the Q_10_ of different ecosystems.

In the current study, SOC content is higher within the southeastern boundary of the QTP than in the Haibei alpine meadow located in the inner QTP^51,88^, and a little higher than in alpine grasslands with an altitude of over 4500 m located in the Tibet^103–105^, but is lower than in the Zoigê alpine wetland located at the eastern edge of the QTP^106,107^. The Rs rate in the alpine wetland meadows in this study is roughly similar to that of degraded grassland located at the northeastern edge of the QTP^108^, but it is higher than in the Haibei alpine meadow^36,51,88,94^ and the inner QTP^11,57,75,76,98^.

The Q_10_ value of the alpine wetland meadow in this study is higher than in the Haibei alpine meadow and other alpine regions (range 1.3–5.6)^36,51,88,94,109–111^, the Zoigê alpine wetland^106,107^, the inner QTP (range 1.05–2.81)^11,75,98^, and the global average (range 1.3–3.3)^85,112^. Many studies have suggested that Q_10_ declines with increasing temperature^51,113–115^. It is worth noting, however, that both the mean temperature and the Q_10_ are higher in this study area than in the Haibei region mentioned above. Furthermore, Q_10_ correlates significantly with SOC in our study (Table 3), but the Q_10_ is higher than in the Zoigê alpine wetland because of the higher SOC content at our study site.

Together, these results suggest that Rs sensitivity to temperature is greater in alpine wetland meadow ecosystems located in the boundary region of the QTP than in other zones. Also, we speculated that Q_10_ of alpine wetland meadow is probable greater at the boundary region than inner region of the Qinghai-Tibet Plateau, and should be a more sensitive indicator in the studying of climate change in this zone.

## Conclusions

To the best of our knowledge, this study is the first to observe Rs on diurnal and seasonal time scales, and to quantitatively analyze Rs and Q_10_ at four different levels of alpine wetland meadow degradation in the Napa Lake region of Shangri-La, at the southeastern edge of the QTP.

In summary, we found that vegetation degradation markedly altered the Rs of the alpine wetland meadow. Rs decreased by more than 50% when degradation intensity increased from NDM to SDM. On the scale of diurnal variation, Rs correlated significantly with Ts at the 0–5 cm soil depth (p < 0.05), but not at the seasonal scale. The Q_10_ value of Rs decreased significantly with an increase in degradation from NDM to SDM during in every season. Rs and Q_10_ were higher during the MGS than during the EGS and LGS at every level of degradation. These results indicate that vegetation condition plays an important role in controlling Rs and Q_10_.

## Materials and Methods

### Site description

**Figure 9:**
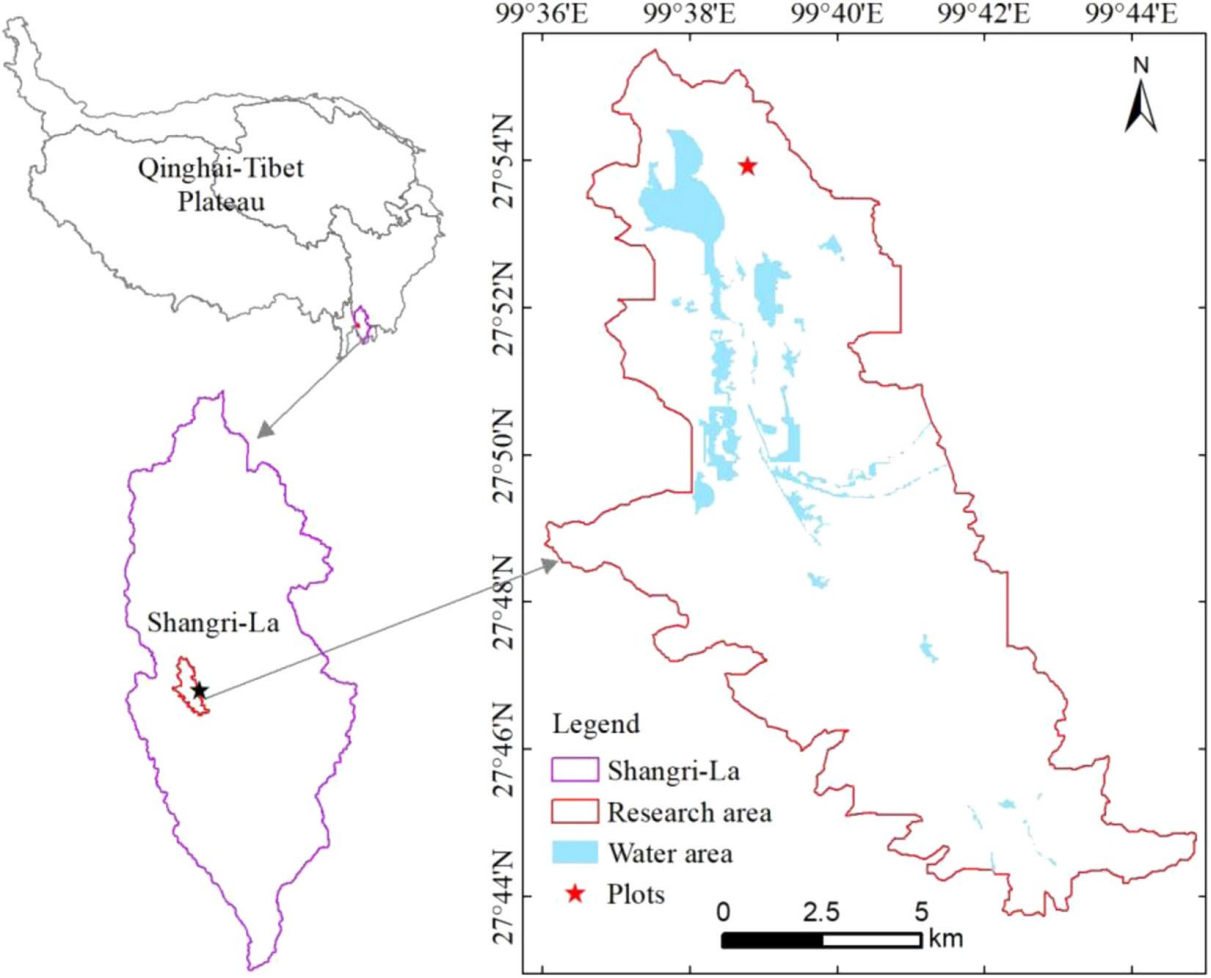
Location of Shangri-La on the Qinghai-Tibet Plateau.

This study was performed at the Napa Lake basin in Shangri-La County (N27°49′–27°55′, E99°37′–99°40′; mean altitude 3350 m), which lies at the southeastern edge of the QTP in northwestern Yunnan province, China (Fig. 9). Napa Lake is a typical plateau lake found on the Yungui plateau. It is situated in a graben basin in the alpine and gorge region of the Hengduan Mountains.

The study region has a cold and moist subtropical southwestern monsoon climate that is influenced by the region’s high altitude and plateau landscape. Mean annual temperature is 6.4 °C, mean monthly minimum and maximum temperatures are −3.6 °C in January and 13.2 °C in July, mean annual precipitation is ~632.4 mm.

The annual range in temperature is small, but the daily range in temperature range is large. The rainy season lasts from June to October and the dry season lasts from November to May. The growing season lasts from about May to September. Soil types in the region are mainly swamp soil, peat soil, and alpine meadow soil.

Vast areas of alpine wetland meadows are distributed around Napa Lake, with dominant plant species including *Blysmus sinocompressus*, *Carex muliensis*, *Poa szechuensis*, *Pedicularis longiflora*, *Kobresia bellardii*, and *Potentilla anserina*. Villages are located far from the lakeside, while alpine meadows and farmland planted with *Hordeum vulgare* are close to the lakeside. As altitude increases, vegetation succeeds gradually from hard-leaf evergreen and broad-leaved forests, to alpine shrubs, to alpine pine forest, and to spruce and fir forests. Dominant species include *Crataegus oresbia*, *Populus rotundifolia*, *Sabina squamata*, *Pinus densata*, *Picea asperata* Mast., and *Abies forrestii*.

### Plot surveys and Rs measurements

#### Study plots

We classified alpine wetland meadows within the study area into four levels of degradation based on the presence of fencing, grazing activity, tourism disturbance, and aboveground biomass and vegetation cover: non-degraded meadow (NDM), lightly-degraded meadow (LDM), moderately-degraded meadow (MDM), and severely-degraded meadow (SDM) (Table 1).

Each of the four levels of alpine wetland meadow degradation severity contained three study plots (100 m × 100 m). All plots are located adjacent to a lake, but are exposed to water year-round, and do not experience periodic flooding. By correlating the degree of degradation in the plots with vegetation cover, biomass, and species composition, we can determine the effects of grazing and tourism on the meadows.

#### Vegetation surveys and soil organic carbon (SOC) measurements

To further characterize and verify the degree of degradation in the different plots, we sampled aboveground biomass, vegetation cover, and species composition within one randomly-placed 1 m × 1 m frame on every plot every season. The leaf area index (LAI) within the sampling frames was determined using a plant canopy analyzer (LI-COR LAI-2200 Plant Canopy Analyzer, Li-Cor, Lincoln, Nebraska, USA).

Plant samples from each sampling frame were dried in an oven at 65 °C for at least 48 h and then weighed. Other plant samples from the sampling frames were combined by plot of same degradation level, dried at 105 °C for 15 min, and then dried at 65 °C for 48 h to measure the carbon content of the vegetation.

One soil profiles with a depth of 50 cm were collected from each plot during the experimental period, and the SOC content of the soil at different depths (0–10 cm, 10–20 cm, 20–30 cm, 30–40 cm, and 40–50 cm) was analyzed for every degradation level.

#### Rs measurement

Rs was measured in each plot using an automated CO_2_ efflux system (Li-8100, LI-COR Inc., Lincoln, NE, USA)^57,68,75^ in May (early growing season, EGS), July (mid growing season, MGS), and September (late growing season, LGS) over the course of the full growing season (May to September) in 2014 and 2015.

CO_2_ measurements were collected from each of the four plots for a period of 24 h using a Li-8100 automated soil CO_2_ flux system with a No. 103 chamber (Li-Cor Inc., Lincoln, NE, USA) to determine diurnal Rs during the growing season in the two years of the study. During the measurements, all chambers were placed on collars with a 20 cm inside diameter and a 10 cm height. The collars had been inserted 5 cm into the soil at least three days prior to measurement. Aboveground vegetation was clipped from the soil surface inside the collars before measuring Rs. All collars were left at the plots during the entire experimental period. Rs include respiration from plant roots and microbes.

Diurnal variations in Rs were recorded automatically every half hour from 7:00 am on the first day to 7:00 am on the next day. The duration of each automatic measurement was about 3 min, which included 15 s dead band, 45 s pre-purge, 45 s post-purge, and 90 s observation. The linear increase in CO_2_ concentration within the chamber was used to estimate Rs. We simultaneously measured Ts and soil moisture at 5 cm soil depth near each collar using the temperature and moisture sensors of the Li-8100 System.

### Statistical analysis

An exponential equation^69^ was used to describe the relationship between Rs and Ts:

1 $${\rm{Rs}}={{\rm{ae}}}^{{\rm{bTs}}}$$

where, Rs is soil respiration rate (μmol·m^−2^·s^−1^), Ts is soil temperature (°C) at 5 cm depth, and a and b are fitted parameters.

The sensitivity of Rs to Ts can be defined as the increase in the Rs rate that results from each 10 °C increase in Ts. This sensitivity (Q_10_) can be calculated as follows:

2 $${{\rm{Q}}}_{10}={{\rm{e}}}^{10{\rm{b}}}$$

One-way ANOVA was used to compare vegetation condition, SOC, and Rs at different levels of degradation. Exponential regression was used to evaluate the relationship between Rs and Ts in every plot. Linear regression was used to correlate vegetation, SOC, Rs, and Q_10_. All statistical analyses were performed using SPSS 13.0 software (SPSS for Windows, Chicago, IL, USA). Differences were considered significant when p < 0.05.

## Data Availability

The datasets generated during and/or analysed during the current study are available from the corresponding author on reasonable request.
